# Do marked discharge summaries with recommendation text boxes enhance patient safety? A nationwide survey

**DOI:** 10.3399/BJGPO.2024.0037

**Published:** 2025-03-12

**Authors:** Thorbjørn H Mikkelsen, Jesper B Nielsen, Maria M Storsveen, Jens Søndergaard

**Affiliations:** 1 Research Unit of General Practice, Department of Public Health, University of Southern Denmark, Odense, Denmark

**Keywords:** patient safety, general practice, discharge summaries, surveys and questionnaires

## Abstract

**Background:**

Danish hospital physicians are required to mark their discharge summaries addressing whether the patient’s GP is recommended to follow-up the patient, as well as suggesting follow-up actions.

**Aim:**

To investigate whether a new form of discharge summaries may contribute to improving perceived patient safety following transition from hospitals to general practice.

**Design & setting:**

A questionnaire sent to a representative sample of GPs in Denmark.

**Method:**

A questionnaire was prepared for GPs based on background material, focus group interviews, and discussions with relevant professionals. It was subsequently pilot tested by fellow researchers and GPs, and revised before the presently reported survey.

**Results:**

Of 310 participating GPs, 197 (63%) ‘totally agree’ or ‘partly agree’ that the marked discharge summaries (MDS) with a recommendation text box contribute to a better handover to general practice, and 223 (72%) ‘totally agree’ or ‘partly agree’ that they improve patient safety.

**Conclusion:**

The majority of responding GPs believe that the MDS with a recommendation text box enhance patient safety and facilitate the transition of care to general practice following hospital discharge.

## How this fits in

Follow-up by the GP after discharge from hospital is important for patient safety and reduces the risk of hospital readmission. Danish hospital physicians are required to mark their discharge summaries addressing whether the GP is recommended to follow-up on the patient as well as to suggest any follow-up action. This study shows that Danish GPs find that the marked discharge summaries (MDS) improve patient safety and the handover to general practice after the patient’s discharge from hospital.

## Introduction

Numerous research studies^1–6^ have examined the communication dynamics between healthcare sectors. Transitions within the healthcare system, such as hospital discharges, can lead to knowledge gaps, misunderstandings among healthcare professionals, ambiguity regarding treatment responsibilities, medication errors, and interruptions in care continuity.^7–10^ Consequently, prioritising effective discharge communication is crucial, particularly in reducing the likelihood of adverse events.^3,11^ In Denmark, the vast majority of patients are registered with a GP who oversees their care and serves as a key intermediary within the healthcare system. Following discharge from the hospital, the patient’s care responsibility shifts from the hospital back to the GP.^12^ Follow-up by the GP after the patient’s discharge from hospital is important for patient safety and reduces the risk of hospital readmission.^3,13^ Yet, GPs often have little time to review discharge summaries,^14–16^ and failures occur in the processing of requested actions in almost half of all discharge summaries.^16–19^

To improve patient safety and handover to general practice, colour-coded discharge summaries have been introduced in Denmark with a brief recommendation to the patient’s GP about how to follow-up on the patient. Since 2019, hospital physicians have been required to mark the discharge summaries if the GP is recommended to follow-up on the patient as well as to state the suggested follow-up action.^20^

The guide regarding the marked discharge summaries (MDS) with a recommendation text box states that:^20,21^

- the hospital physician must mark the discharge summaries in which they advise on the specific follow-up by the patient’s GP;
- the hospital physician must fill in the recommendation text box on the MDS;
- within given time limits (see time limits in Textbox 1), GPs must review discharge summaries marked as containing a recommendation for follow-up on discharge.

In addition, the Danish Regions and Organisation of General Practitioners (PLO) in Denmark agreed in their collective agreement for 2018 that the MDS should be colour marked with green, yellow, or red.^22^

### Textbox 1: The meaning of the colours of the discharge summaries

The discharge summaries should be marked in accordance with the following principles:^23^

- red colour marking: when the patient needs follow-up in general practice within 1–2 working days after discharge from hospital. When red marking is used, the hospital phones the patient’s GP;
- yellow colour marking: when the patient is vulnerable* and not expected on their own to contact their GP after discharge from hospital, and has a particular need for active follow-up in general practice within 14 working days;
- green colour marking: other discharge summaries that contain recommendations on non-urgent follow-up in general practice;
- no colour marking: discharge summaries without recommended follow-up.

* 'Vulnerable' patients in this context refers to individuals at risk of readmission, those grappling with severe illnesses, multiple concurrent conditions necessitating treatment, disabilities, and potentially limited personal networks. These patients heavily rely on health and social services, often lacking robust personal resources and facing challenges related to their understanding of illness, social environment, or cultural factors. As a result, they may struggle to engage in specific behaviours and self-care practices.^24^

It should be noted that the red, yellow, or green colour codes will not necessarily be displayed in the GPs’ electronic medical record (EMR) system but may — depending on the user interface of the EMR — be described in text only.

Discharge summaries with a recommendation text box is not a novelty.^25^ However, to our knowledge, in 2019, Denmark became the first country to implement MDS with a recommendation text box. Therefore, it is important to examine if the MDS is perceived as supporting GPs’ work and patient safety. This study did not present a particular definition of patient safety but draws on the context of and on the authorities’ use of the notion in their communication regarding the MDS including:

*'The purpose of the discharge summary guidelines is to achieve a more patient-safe transfer from hospital to the GP. Patient safety is strengthened when discharge summaries are easy to manage. This will be done by both having a recommendation field at the top and by making it more manageable for their GP which discharge summaries contain a recommendation for follow-up. Therefore, the guidelines require that information about the need for follow-up is clear and at the top of the discharge summary when it is received by the patient’s GP.'* ^20^

This study aims to investigate the GP’s experience with the MDS scheme with emphasis on the transition from hospital to the GP as well as patient safety.

## Method

### Study population

A questionnaire was sent to a representative sample of GPs in Denmark by the PLO, which is the professional body for all GPs in Denmark. The questionnaire survey was carried out from 5 January 2021 until 26 January 2021. Non-responders received one reminder after 2 weeks. It took approximately 15 minutes to complete the questionnaire. The GPs were remunerated in accordance with instructions laid down by the Danish Society of General Practitioners (DSAM), corresponding to DKK 206.43 (approximately 23.50 GBP) for answering the questionnaire.

The questionnaire was distributed and collected by the PLO, which dispatched it to 800 randomly chosen GPs listed in the PLO’s membership databases holding >99% of all Danish GPs. Two were deleted immediately owing to inactive mailboxes, leaving 798 as presented in Table 1. In addition, one was removed owing to participation in this project and one more who was not working in general practice at that time leaving 796 eligible GPs. This approach ensured an equitable distribution across the entire group in terms of sex, age, region, and practice type. At the time of the survey, PLO’s membership databases contained 3473 GPs showing that approximately one-quarter was invited. As can be seen from Table 1, the sample and the responders closely resemble the composition of Danish GPs regarding sex, age, and geographic region.

**Table 1.** Characteristics of responding GPs (*n* = 310) compared with all Danish GPs

	Poulation of Danish GPs		Sample of GPs		Responding GPs	
	*n*	%	*n*	%	*n*	%
**Sex**						
Male	1499	43	340	43	144	46
Female	1974	57	458	57	166	54
**Geographic region**						
The Capital Region of Denmark	1064	31	248	31	88	28
The Central Denmark Region	441	13	87	11	41	13
The North Denmark Region	819	24	200	25	79	25
Region Zealand	851	25	197	25	78	25
Region of Southern Denmark	298	9	66	8	24	8
**Age, years**						
30–40	320	9	81	10	30	10
41–50	1421	41	316	40	119	38
51–60	989	28	229	29	95	31
61–70	660	19	154	19	61	20
≥70	83	2	18	2	5	2

### Questionnaire

We prepared the questionnaire for the GPs based on background material, focus group interviews, and discussions with the project advisory board, including representatives from Danish Regions, PLO, MedCom and The Danish Patient Safety Authority. Constructed with answers on a 5-point Likert scale, the questionnaire was pilot tested by fellow researchers and GPs, and revised before the survey (see supplementary file for final questionnaire).

### Data analysis

Each region has its own EMR in which the discharge summaries are made. However, Region Zealand and the Capital Region have the same EMRs. The GPs were using eight different EMRs, each of which displays the discharge summaries in different ways. These aspects, in combination with personal preferences, such as the type of clinic and other aspects, may influence the GPs’ perceptions of the changes in the discharge summaries. Both unadjusted and adjusted analyses were carried out. We adjusted for the influence of region, EMR, sex, age group (aged ≤55 years versus aged >55 years), type of clinic (solo versus multi), and whether the clinic employs nurses. The rationale for including the list of covariates in the adjusted multivariate models is that sex and age could possibly affect the GPs' willingness to embrace new electronic solutions. The size of the clinic and whether it employs nurses may influence how used to the GPs are to taking advice from colleagues and hence how they perceive recommendations from other healthcare professionals. We applied an adjusted logistic regression, adjusting for geographic region, software used by the clinics, sex, age group, type of clinic, and whether the clinic employed one or more nurses. A *P* value of ≤0.05 was considered statistically significant.

## Results

The sex distribution among those who answered the questionnaire is largely similar to the age and sex distribution among all GPs, and the distribution of the responses is similar to the distribution of GPs in the regions (Table 1).

The distribution of software is approximately the same among Danish GP clinics and among the responding GPs (Table 2).

**Table 2.** The distribution of software in general practice clinics and among the GPs who have answered the questionnaire

	Distribution of software among Danish GP clinics		Distribution of software among responding GPs	
**Software used by the clinics**	*n*	%	*n*	%
MDS 1	725	42	133	43
MDS 2	436	25	87	28
MDS 3	201	12	27	9
MDS 4	239	14	46	15
MDS 5, MDS 6, MDS 7^a^	114	7	17	5
MDS 8	1	0	0	0

^a^MDS 5, MDS 6, MDS 7 are merged owing to low number of users. MDS 1 to MDS 8 = software used by the GP clinics.

Of the 310 participating GPs 197 (63%) ‘totally agree’ or ‘partly agree’ that the MDS contribute to a better handover to general practice, while 68 (22%) ‘neither agree nor disagree’, and 25 (8%) and 20 (6%), respectively, ‘partly disagree’ and ‘totally disagree’ (Table 3). When asked if the MDS with the recommendation text box improve patient safety, 223 (72%) ‘totally agree’ or ‘partly agree’ that the MDS improve patient safety; 45 (15%) ‘neither agree nor disagree’, while 24 (8%) ‘partly disagree’, and 18 (6%) ‘totally disagree’ (Table 3).

**Table 3.** Improved handover and patient safety

	Totally agree	Partly agree	Neither agree nor disagree	Partially disagree	Totally disagree	
	*n* (%)	*n (*%)	*n* (%)	*n* (%)	*n* (%)	*n*
The new MDS with a recommendation text box contribute to a better handover to general practice	63 (20)	134 (43)	68 (22)	25 (8)	20 (6)	310
The new MDS with a recommendation text box improve patient safety	67 (22)	156 (50)	45 (15)	24 (8)	18 (6)	310

MDS = marked discharge summaries

After adjusting for geographical region, sex, age groups, type of clinic, or whether the clinic employs nurses, there were no statistical differences in the perception of whether the MDS contribute to a better handover to general practice, except from the rarely used software of MDS 5, MDS 6, MDS 7. These three programs, which were merged in the analysis owing to a low number of users, showed a statistically significant poorer handover than the other types of software used by the GPs (Table 4).

**Table 4.** The new, marked discharge summaries with a recommendation text box contribute to a better handover to general practice

	Prev	‘Totally agree’ or ‘partly agree’	‘Neither agree nor disagree’, ‘partially disagree’ or ‘totally disagree’	OR (95%CI)^a^	Adjusted OR (95%CI)^b^
		*n* (%)	*n* (%)		
**Geographical region**					
The Central Denmark Region	78	55 (70.5%)	23 (29.5%)	1	1
The Capital Region of Denmark	88	64 (72.7%)	24 (27.3%)	1.12 (0.57 to 2.19)	1.28 (0.61 to 2.68)
The North Denmark Region	24	16 (66.7%)	8 (33.3%)	0.84 (0.31 to 2.23)	0.82 (0.30 to 2.28)
Region Zealand	41	26 (63.4%)	15 (36.6%)	0.72 (0.33 to 1.62)	0.92 (0.39 to 2.14)
Region of Southern Denmark	79	62 (78.5%)	17 (21.5%)	1.53 (0.74 to 3.15)	1.55 (0.74 to 3.22)
**Software used by the clinics**					
MDS 1	133	102 (76.7%)	31 (23.3%)	1	1
MDS 2	87	62 (71.3%)	25 (28.7%)	0.75 (0.41 to 1.39)	0.81 (0.43 to 1.54)
MDS 3	27	17 (63.0%)	10 (37.0%)	0.52 (0.21 to 1.25)	0.63 (0.24 to 1.68)
MDS 4	46	35 (76.1%)	11 (23.9%)	0.97 (0.44 to 2.13)	0.91 (0.38 to 2.19)
MDS 5, MDS 6, MDS 7^c^	17	7( 41.2%)	10 (58.8%)	**0.21 (0.07 to 0.61)^d^**	**0.29 (0.09 to 0.92)^e^**
**Sex**					
Male	144	96 (66.7%)	48 (33.3%)	1	1
Female	166	127 (76.5%)	39 (23.5%)	1.63 (0.99 to 2.68)	1.43 (0.84 to 2.41)
**Age, years**					
≤55	194	146 (75.3%)	48 (24.7%)	1	1
>55	116	77 (66.4%)	39 (33.6%)	0.65 (0.39 to 1.08)	0.77 (0.44 to 1.34)
**Type of clinic**					
Multi^f^	235	178 (75.7%)	57 (24.3%)	1	1
Solo^g^	75	45 (60.0%)	30 (40.0%)	**0.48 (0.28 to 0.83)^h^**	0.63 (0.34 to 1.18)
**One or more nurses employed in the clinic?**					
Yes	275	199 (72.4%)	76 (27.6%)	1	1
No	35	24 (68.6%)	11 (31.4%)	0.83 (0.39 to 1.79)	1.07 (0.45 to 2.53)

^a^Crude prevalence ratio is the unadjusted prevalence ratio. ^b^Adjusted logistic regression. Adjusted for geographic region, software used by the clinics, sex, age group, type of clinic, and whether the clinic employs one or more nurses.^c^MDS 5, MDS 6, MDS 7 are merged owing to few users. ^d^ *P* = 0.004. ^e^ *P* = 0.036. ^f^Partnership clinics. ^g^Solo, shared single, and groups of solo GPs sharing clinic facilities. ^h^ *P* = 0.009. MDS 1 to MDS 8 = software used by the GP clinics. Prev = prevalence.

Table 5 shows that in the perception of whether the MDS improve patient safety, there are no statistical differences between geographical region, software used by the clinics, sex, age groups, type of clinic, or whether the clinic employs nurses.

**Table 5.** The new marked discharge summaries with a recommendation text box improve patient safety

	Prevalence	'Agree' or 'partly agree'	'Neither agree nor disagree', 'partially disagree', or 'totally disagree'
		*n* (%)	*n* (%)
**Geographical region**			
The Central Denmark Region	78	47 (60.3%)	31 (39.7%)
The Capital Region of Denmark	88	58 (65.9%)	30 (34.1%)
The North Denmark Region	24	13 (54.2%)	11 (45.8%)
Region Zealand	41	23 (56.1%)	18 (43.9%)
Region of Southern Denmark	79	56 (70.9%)	23 (29.1%)
**Software used by the clinics**			
MDS 1	133	89 (66.9%)	44 (33.1%)
MDS 2	87	54 (62.1%)	33 (37.9%)
MDS 3	27	17 (63.0%)	10 (37.0%)
MDS 4	46	31 (67.4%)	15 (32.6%)
MDS 5, MDS 6, MDS 7^a^	17	6 (35.3%)	11 (64.7%)
**Sex**			
Male	144	84 (58.3%)	60 (41.7%)
Female	166	113 (68.1%)	53 (31.9%)
**Age, years**			
≤55	194	129 (66.5%)	65 (33.5%)
>55	116	68 (58.6%)	48 (41.4%)
**Type of clinic**			
Multi^b^	235	158 (67.2%)	77 (32.8%)
Solo^c^	75	39 (52.0%)	36 (48.0%)
**One or more nurses employed in the clinic?**			
Yes	275	177 (64.4%)	98 (35.6%)
No	35	20 (57.1%)	15 (42.9%)

^a^MDS 5, MDS 6, MDS 7 are merged owing to few users. ^b^Partnership clinics. ^c^Solo, shared single, and groups of solo GPs sharing clinic facilities. MDS 1 to MDS 8 = software used by the GP clinics

## Discussion

### Summary

This study shows that the GPs perceive that the MDS improve both patient safety and the handover to general practice after the patients have been discharged from hospital. Besides the software used in the clinic, no other clinic characteristics appeared to significantly affect usability of the recommendation text box. The MDS contribute to reliable discharge summaries, an improvement previously called for elsewhere.^15^ The few GPs using the software of MDS 5, MDS 6, and MDS 7, which were merged owing to the low number of users in our analysis, experienced a statistically significant poorer handover to general practice than the other types of software (*P* = 0.036). While these results apply to few GPs, it is unclear whether the differences are owing to EMRs not being adjusted to display to the new MDS properly.

### Strengths and limitations

The purpose of the MDS is to ensure better knowledge transfer to general practice and improved patient safety. This questionnaire does not provide an unambiguous answer to the question, as we have only asked about the GPs’ experiences. However, we consider it a strength of this study that it has gained data about the experiences from the target group of the discharge summaries: the GPs. It is also considered a strength that the sample and the responders closely resemble the composition of Danish GPs. The response rate of 38.8% is a limitation to this study but is not uncommon in surveys of general practice.^26–28^ This may lead to biased results, since GPs who feel the strongest about the new discharge summaries are probably those most inclined to answer the questionnaire, regardless of whether they like or dislike the changes.

### Comparison with existing literature

Improved communication tools between hospital physicians and GPs have often been requested.^4–6^ Discharge summaries with a recommendation text box is not a novelty.^25^ However, to our knowledge, Denmark was the first country to implement the MDS including a recommendation text box. The results are positive, showing that the MDS ensure that the GPs know which discharge summaries to act on immediately after the patient has been discharged, thus reducing information overload.

Priority flags and priority scanning have been suggested as possible improvements in GP clinics.^16^ The discharge summaries with the recommended text boxes seem to have created a solution where the hospital physician helps the GP make the initial flag indicating which patients they think the GP should prioritise.

Studies have shown that an increased risk of failure to process test requests in discharge summaries was associated with the discharge summaries.^16^ These aspects could also have influenced this study and caused a difference in the results between the regions as well as the EMR used by the GPs. However, the variations in our results are not statistically significant after adjustment.

Studies have indicated the importance of follow-up strategies in ensuring a smooth transition to the GP.^2,3,16,19,29,30^ This research further emphasises that GPs perceive the MDS implemented in Denmark as enhancing the handover process to general practice and improving patient safety. Consistent with recommendations from other sources, hospitals are encouraged to pursue standardisation in the design of discharge summaries.^16^ While authorities in Denmark delineate the structure of discharge summaries, individual geographic regions maintain autonomy over their EMR systems.^21,31^ Our findings highlight some variations across different regions. Region Zealand and the Capital Region use the same EMR.^32^ Hence, it could be expected that the results would be somehow similar. However, in the Capital Region 64 (72.7%) agree or partly agree that the new discharge summaries improve the handover, compared with the 26 (63.4%) in Region Zealand. Regarding patient safety, the numbers are 58 (65.9%) and 23 (56.1%), respectively. While the differences are statistically insignificant, they indicate that the results within the two regions are being influenced by other aspects. Since the EMR is the same, the differences might be caused by the EMR being used differently. Automatically generated codes that may only be used if they are relevant for the GP^21,33^ might account for some of the difference if not used properly.^3^

### Implications for research and practice

The survey shows that the GPs perceive that the MDS improve patient safety and the handover to general practice after the patient has been discharged from hospital. It also shows that the differences between the regions and the EMRs used by the GPs are relatively large, but not statistically significant on an overall level. Future studies should explore the GPs’ experiences with the new discharge summaries in further detail, for example, subsequent readmission rates.

## Declarations

The study was recommended by the Multi-Practice Committee of the Danish Society of General Practitioners and The Organization of General Practitioners in Denmark (MPU 29-2019).

The study was conducted in accordance with guidelines for good scientific practice.

Storage management of the data fulfilled the European General Data Protection Regulations (GDPR) and was registered with the Research and Innovation Organization (RIO), University of Southern Denmark (Project number 10.795).

## Data

The datasets are not publicly available due to regulations from The Danish Data Protection Agency.
